# Review

**Published:** 1839-06

**Authors:** Solyman Brown


### REVIEW,

BY SOLYMAN BROWN.

"The Dental Art, a practical treatise on Dental Surgery, by Chapin A. Harris, M. D., Surgeon Dentist."—Baltimore—Armstrong & Berry, 1839, 338 pp. Octavo.

The members of the profession, and students in particular, have long felt the necessity of a treatise on Dentistry in all its branches, medical, surgical and mechanical, which should at once be comprehensive in its scope, practical in its detail, correct in its science, and beautiful in its typographical execution. This desideratum is now supplied in a manner highly creditable to the accomplished author, and peculiarly acceptable to the profession at large. No dental work of exactly a similar character has ever been issued by the American press, and thence it comes in competition with the work of no living author on this side of the Atlantic. In comparison with English and French publications on the same subject, it can suffer nothing, and indeed possesses one advantage, inasmuch as it brings down the history and knowledge of our art to the present day, with all the improvements which the genius of American practitioners has introduced into their favorite science.

Dr. Harris has proved himself so well qualified for his task, that it may be safely asserted, that no one volume in the English language cantains an equal amount of correct and valuable information for the use of the student in dentistry; and indeed the pupil would be compelled to ransack many volumes in different languages to arrive at all the valuable truths comprised in this work. There are manifested also in Dr. Harris' volume, an originality of thought, and an independence of opinion equally calculated to elicit truth and expose error.

But there is still another point of view in which this treatise should be regarded, by all who are either operators or subjects of dental practice. It is this:—The author has so well acquitted himself, that his work is calculated to give dignity and importance to the subject of which it treats; evincing that the writer is one of those high-minded men who impart respectability to the calling which they pursue, rather than increase their own importance by its exercise.

The time has come when the Medical Faculty, and the most discerning members of society at large, are beginning to rank the profession of the Dentist with those other avocations of men, which are honourable because they are useful, and profitable, because their exercise demands peculiar qualifications to ensure success.

In all professions, there will be found those who presume upon abilities which others never discover, and wonder that fortune does not smile upon their presumption. But the period is not far distant, when it will be learned, as well by the profession as by its patrons, that if there are any arts of civilized society which require for their exercise, education, genius, moral integrity, solid judgment, scientific discrimination, and mechanical skill, the Art of Dentistry stands pre-eminent among the number.

Who would willingly permit a stupid bungler, ignorant alike of his own business and of every thing else, to operate upon the delicate and sensitive organs of the mouth? Whatever qualifications other callings may require, most assuredly, the offices of the Dental Operator should be performed by a gentleman in manners, an artist in skill, a man of tender sensibility and refined decorum.

It is surely no flattering compliment, either to the profession which we follow, or to the community which requires its services, that in many instances, those who have been openly disgraced by notorious criminality, are still the objects of public favour. For the correction of this, and many other evils, publications like that under review, are eminently designed. Dentistry, with its present improvements, is a new science, and it requires writers of education and genius to give it consistency and reputation.

This work contains no irrelevant matter; every page is filled with useful and interesting information; so that the student will find it a text-book exactly suited to his wants, while the general reader, or the educated dentist, without meeting with any thing to offend his taste, will find something to increase his knowledge.

The following extracts will furnish the reader with a specimen of the author's style; but we trust that every member of the profession will avail himself, without delay, of the entire work.

### ABUSES OF DFNTISTRY.

"Confided, as this branch of surgery always has been, in almost every country, to the hands of whoever seemed disposed to attempt the exercise of its intricate duties, it is not to be wondered, that it should have frequently fallen into obloquy and disrepute. But how much soever it may have suffered from this cause, its utility is not the less great, and we can only regret that public opinion has not been sufficiently awakened to its importance to defend it from the impositions of pretending empirics.

It is a remarkable and humiliating fact, that though dental surgery was never better understood, yet its principles were never more erroneously practiced and shamefully abused than at the present day. It may now be said to have attained its greatest perfection; but while there are many that have carefully studied its principles, and devoted themselves with zeal and integrity to its practice, there are others, actuated by less praiseworthy motives, that have attempted to discharge its duties without possessing any of the necessary preparatory qualifications, and in consequence, many injuries have resulted from their operations."

The author has the following remarks on the effects of Mercury:

"Mercury does not, as many imagine, exert any direct action on the teeth; it is only by the effects that it sometimes produces in the gums and the secretions of the mouth, that they are injured by its use. When it is given in sufficient quantities, and long enough to produce ptyalism, however slight, it becomes hurtful to the teeth, and just in proportion as it affects the juices of the mouth, are the corrosive properties of these fluids increased. Hence, it can be considered only as an indirect cause in the production of caries.

The relation which the teeth sustain to the maxillæ, however, is often very seriously affected, and sometimes entirely destroyed, by the exhibition of this medicine. Its introduction into the system is generally followed by an increased action of the absorbents, and in no part of the body is this more evident than in the gums and alveolar processes. It sometimes occasions a very rapid loss of substance in these parts, so that the teeth, by the absorption of their sockets, are loosened, and, in a few months, caused to drop out. A few years ago, an application was made to me, for a set of artificial teeth and gums, by an interesting young lady, of only twenty years of age, who had lost nearly the whole of her teeth, by the absorption of her gums and alveolar processes. She informed me, that about four years before, she had been afflicted with a severe attack of bilious fever, and during its continuance, had taken a great deal of mercury, and, in consequence, had been so badly salivated, that her teeth were loosened, and soon after her recovery, dropped out one after another, in spite of the efforts of several physicians and dentists to preserve them, until only nine remained.

The deposition of tartar upon the teeth, is much increased by the use of this medicine, especially when it affects the saliva. So much, in fact, is the tendency to a deposition of this substance increased by its exhibition, that persons laboring, for the first time, under a mercurial diathesis, frequently have the crowns of their teeth, opposite the mouths of the salivary ducts, completely coated with it in a few days."

Remarks while treating of Mechanical operations.

" Many have chosen dentistry as a profession, with the belief that a knowledge of it was more easily acquired, than that of any other; and some, after having followed it for two or three years, and finding that in order to attain respectability and usefulness in it, greater difficulties were to be surmounted than they had anticipated, have preferred to abandon it altogether, rather than bring disgrace upon both it and themselves. Let no one, therefore, be deceived into a belief, that, in a few weeks or months, he can become master of the art; for, should he commence its study under such an impression, he will most assuredly be disappointed, and, perhaps, find, after having devoted to it what he before thought sufficient time for its entire acquisition, that he has scarcely attained to a knowledge of its elementary principles."

